# Editorial: International Perspectives on Mental Health and Mental Health Social Work

**DOI:** 10.3390/ijerph19127387

**Published:** 2022-06-16

**Authors:** Jim Campbell, Lisa Brophy, Gavin Davidson

**Affiliations:** 1 School of Social Policy, Social Work and Social Justice, University College Dublin, D04 V1W8 Dublin, Ireland; 2 Social Work & Social Policy, La Trobe University, Melbourne 3086, Australia; l.brophy@latrobe.edu.au; 3 School of Social Sciences, Education and Social Work, Queen’s University Belfast, Belfast BT7 1NN, UK; g.davidson@qub.ac.uk

## 1. Introduction

The following collection of articles reflects the diversity of research, policy and practice in mental health social work in a range of international contexts. We will set the scene for this Special Issue by reflecting upon the origins of the profession and the various challenges and opportunities that affect contemporary mental health social work practice across the world. The editorial will conclude with an appeal for the profession to embrace new approaches, some of which are reflected in the collection, to enable mental health social workers to engage in more empowering ways with individuals, families and communities.

## 2. The Origins of Mental Health Social Work

The development of the profession of social work, especially in the UK and the USA, can be traced to Victorian philanthropy, shaped by the activities and ideas of Mary Richmond, Jane Adams, and other reformers. The movements they led were informed by the assumption that, through inquisitive examination, important issues of disadvantage could be revealed, and social change achieved. The specialism and knowledge base of mental health social work that was developed during the twentieth century evolved to encompass the ideas of Freud and post-Freudians, followed by humanist and behavioural paradigms [1]. Practice was generally underpinned by an optimism that by developing forms of care and treatment, the lives of people with mental health problems could be improved [2].

## 3. The Voice of Service Users

The assumption, however, that mental health services are rationally delivered and necessarily benefit citizens has been challenged in recent decades. A range of critical professional voices have questioned this sense of progress and the effectiveness of mental health services [3,4,5]. It has been argued, for example, that decisions made by mental health professionals can lead to forms of iatrogenic harm, e.g., where medication has excessive side effects or compulsory treatment and hospitalisation are used. There are also concerns that mental health services create or reinforce systems of power and inequality which tend to disadvantage those who receive the services. This is partly achieved in the way that ‘experts’, such as mental health social workers, construct and use exclusive forms of knowledge, as well as language which often reduces the space for service users’ rights and lived experiences to be acknowledged [6]. Partly as a response to these criticisms, new policy commitments to recovery-based approaches in many jurisdictions around the world have evolved. The literature suggests that by embracing such approaches, mental health social workers and others may partner with service users to create opportunities for change and control in their lives, but on their terms. Although there are diverse approaches to recovery that involve competing models, the key principles, including hope and empowerment, are generally agreed. Notions of recovery are often associated with appeals to strengthen the human rights of service users and families, and to reform mental health services worldwide [7]. In response to these developments, expectations about the role of mental health social workers have shifted away from older, paternalistic attitudes and decision-making processes to more empowering positions, where the views of services users and their families should authentically be listened to and acted upon [8].

## 4. Contemporary Practice Issues

The role of the mental health social worker involves complex decision-making processes, often in multidisciplinary settings where casework approaches tend to be prioritised [9]. Mental health social workers, alongside other professionals, often intervene in the lives of citizens when they are at risk because of their mental ill-health. The literature highlights the competing paradigms that inform or influence professional decision making in this area [10]. Professionals are familiar with actuarial and clinical approaches to risk assessment, but less so about the argument that is often made by mental health advocacy organisations, where there is a focus on the risks that service users can experience caused by professionals and mental health services [11].

There is a growing expectation that mental health professionals should consider alternative perspectives that highlight how structural issues, such as poverty, racism and gendered inequalities, define the lives of people with mental health problems. It has also been argued that Eurocentric paradigms fail to understand alternative, international social work modalities that often vary between cultures and international jurisdictions [12]. Although a high proportion of a mental health social worker’s time is spent working collaboratively with clients, there are moments when coercive, controlling interventions are used. In many jurisdictions, mental health social workers, alongside medical practitioners, can compulsorily admit service users to hospitals and other facilities against their wishes, even when service users can make the relevant decisions. There are competing debates about the purpose and nature of the decision-making processes that are associated with these roles. A conventional view is that there is, at times, a need to protect vulnerable service users and others at times of crisis in the application of the principles of *parens patriae*. In responding to the principles of the United Nations Convention on the Rights of People with Disabilities (UNCRPD), States are now required to move away from the traditional type of substitute decision making and best interests approaches described above, towards supported decision-making processes, where principles of rights, will and preferences are privileged [13].

## 5. The Collection of Articles

These themes are variously reflected in the papers now summarised. We are pleased that the articles analyse and discuss issues of mental health and practice in different parts of the world, using a diverse range of methodologies. The theme of international comparison in the mental health social work role is considered by Stone and colleagues, social work academics in the UK, presenting the findings from an exploratory survey of mental health social work practitioners and researchers in Europe. Although there has been some previous work on social work in Europe, perhaps most notably by Walter Lorenz, such international comparison, especially in specific areas of practice, is still relatively rare. The survey aimed to develop an understanding of the similarities and differences in mental health social work, between and within a range of countries in Europe. It was conducted online, and people were invited to participate through existing networks, such as the European Social Work Research Association (ESWRA) and social media. There were 158 responses from 10 countries and, although this is far from representative of all mental health social work in Europe, it does provide a helpful overview of some of the main themes. These included the importance and distinctive contribution of social work in mental health services, as well as the variations in the balance of different aspects of the role, including therapeutic, legal and specialist/generic approaches. Some of the limitations of the survey are acknowledged, especially around the need for multilingual approaches and better developed networks for ongoing exchange and learning. The article demonstrates the benefits and complexities of international comparison and reinforces the need for further work in this area.

In the second article by Regehr and colleagues, again drawing upon international comparisons, experiences from Canada, Israel and the US are explored, presenting an ecological model for decision making about risk in mental health services. Assessing the potential risk of harm to self and/or others is arguably one of the most difficult aspects of mental health social work practice, especially in situations of high-stress and when the potential consequences include compulsory intervention and/or avoidable harm. This ecological model was developed based on qualitative survey data and experimental designs in each of the authors’ countries. It suggests that standardised approaches to risk assessment are limited as they tend to focus on client factors and may neglect the importance of the professional decision-making processes. These may be influenced by the professionals’ experiences, the relationships involved and the wider organisational, policy and societal context of the assessment. The authors suggest that professionals should explore all of the factors involved in these decision-making processes through developing greater awareness and using dialectical reflection. They further argue that change is also needed at the organizational and policy levels to develop guidance and frameworks which support this more process and context-focused approach to decision making.

In their article, a group of social work educators in Australia, led by Louise Whitaker, have critically reflected on paradigms that currently frame mental health social work education. The paper compares and contrasts the influences of neo-liberalism, critical theory, human rights and post-structuralism on mental health social work practice. They have identified opportunities for improvement and innovation that might be addressed in the context of transformative paradigms. This article has the potential to support emerging practitioners to increase their preparedness to challenge the dominance of the biomedical approach and to sustainably contribute to fostering social justice and human rights in their mental health workplaces. The authors acknowledge that this transformation begins in the classroom, and requires reinforcement in field education and support through the effective supervision of emerging practitioners and communities of practice.

We now move to another part of the world, Hong Kong, for our next article which explores the important intersections between issues of social work practice and political conflict. The authors, Siu-ming To and colleagues, used a cross-sectional survey to explore how political issues impacted on the mental health of 1330 secondary students. Employing an explanatory socio-ecological perspective, they considered how stress caused by political circumstances, intrapersonal, interpersonal and community factors appeared to have affected the lives of young people. A multiple regression analysis revealed direct and moderation effects, including low levels of meaningfulness in life, resilience, social support, youth empowerment in the community, and high civic engagement in the community. The authors conclude that such findings can be viewed as important in developing interventions and programmes that will enhance the lives of young people in Hong Kong in such precarious political situations.

The importance of family approaches to recovery in mental health services is forefront of the literature review by Michael John and Kerry Cuskelly. They begin by highlighting how notions of recovery have become important in creating organisational and cultural changes in how services are structured and delivered. As discussed in the editorial for this Special Issue, recovery involves the service user living the best life of their choice, despite the presence of mental health challenges. The authors point out, however, that relatively little is known about ideas of recovery in family contexts even though there may be unique levels of support that would enable families to embark on their own recovery journeys. The paper seeks to remedy this lacuna. Following a systematic review of the literature they analysed three studies, reflecting issues of family recovery interventions across the lifespan. The benefits and challenges of each intervention to the family were synthesised along with a list of four family-recovery enablers that are vital for the implementation of such interventions.

The final article in this collection focuses on another area of mental health, this time from a Spanish perspective. Rodriguez-Besteiro and colleagues sought to explore the gendered impact of COVID-19 on university students. Three hundred students completed an online questionnaire which analysed variables of perceived risk, psychological profiles, and nutritional, oral health, and physical activity habits. They found that women expressed a greater sense of danger than men. Although there are complexities and ongoing debates about these concepts and how to measure them, the scores also indicated that women reported higher levels of anxiety, conscientiousness, neuroticism, and openness to experience, while men presented higher levels of extraversion. In terms of nutrition, men were more likely to consume soft drinks, meat, and pasta or rice, and had lower buccal hygiene, but no differences were found in terms of physical activity patterns. The scope of the study is an important reminder of the importance of the complex interactions between mental and physical health, which is perhaps an aspect of mental health social work that should be further developed. The authors conclude by suggesting that such findings might be of importance for educational institutions to design interventions to reduce stress and risk perception in the lives of their students.

## 6. Looking to the Future

Each of the articles in this collection highlight challenges for practitioners and researchers and raise questions about how we should consider the role of mental health social workers and other professionals in addressing new and yet to be realised challenges. The range of papers reveal how important it is to see policy and practice in their international contexts, and that no one country paradigm can fully embrace the complex nature of mental health interventions when they are applied to diverse populations. It can be argued, however, that a universal, human rights approach can be, in some ways, modified for all jurisdictions and to insist that the voice of service users be heard; this seems even more important when events such as the pandemic and local and international political conflicts impact on services. Perhaps most importantly, we argue that these papers support the ongoing transition to more inclusive, equitable, rights-based approaches which can help to better understand and respond to the needs of people with mental health problems. Instead of reverting to traditional, subject–object perspectives on the relations between the professional and the service user, where power is generally located with the expert, we should be more prepared to look to alternative visions of mental health and ill-health. This is where the site of intervention becomes focused on the enhancement of population-level life chances and thus citizenship rights for all [14].
